# FMRFamide-Like Peptide 22 Influences the Head Movement, Host Finding, and Infection of *Heterodera glycines*

**DOI:** 10.3389/fpls.2021.673354

**Published:** 2021-06-22

**Authors:** Jia You, Fengjuan Pan, Shuo Wang, Yu Wang, Yanfeng Hu

**Affiliations:** ^1^Key Laboratory of Soybean Molecular Design Breeding, Northeast Institute of Geography and Agroecology, Chinese Academy of Sciences, Harbin, China; ^2^Institute of Pratacultural Science, Heilongjiang Academy of Agricultural Science, Harbin, China; ^3^College of Agricultural Resource and Environment, Heilongjiang University, Harbin, China

**Keywords:** *Heterodera glycines*, FMRFamide-like peptides, neuropeptides, soybean cyst nematode, soybean

## Abstract

The FMRFamide-like peptides (FLPs) represent the largest family of nematode neuropeptides and are involved in multiple parasitic activities. The immunoreactivity to FMRFamide within the nervous system of *Heterodera glycines*, the most economically damaging parasite of soybean [*Glycine max* L. (Merr)], has been reported in previous research. However, the family of genes encoding FLPs of *H. glycines* were not identified and functionally characterized. In this study, an FLP encoding gene *Hg-flp-22* was cloned from *H. glycines*, and its functional characterization was uncovered by using *in vitro* RNA interference and application of synthetic peptides. Bioinformatics analysis showed that *flp-22* is widely expressed in multiple nematode species, where they encode the highly conserved KWMRFamide motifs. Quantitative real-time (qRT)-PCR results revealed that *Hg-flp-22* was highly expressed in the infective second-stage juveniles (J2s) and adult males. Silencing of *Hg-flp-22* resulted in the reduced movement of J2s to the host root and reduced penetration ability, as well as a reduction in their subsequent number of females. Behavior and infection assays demonstrated that application of synthetic peptides Hg-FLP-22b (TPQGKWMRFa) and Hg-FLP-22c (KMAIEGGKWVRFa) significantly increased the head movement frequency and host invasion abilities in *H. glycines* but not in *Meloidogyne incognita*. In addition, the number of *H. glycines* females on the host roots was found to be significantly higher in Hg-FLP-22b treated nematodes than the ddH_2_O-treated control J2s. These results presented in this study elucidated that *Hg-flp-22* plays a role in regulating locomotion and infection of *H. glycines*. This suggests the potential of FLP signaling as putative control targets for *H. glycines* in soybean production.

## Introduction

Soybean [*Glycine max* L. (Merr)] is an important and frequently consumed crop and serves as a major source of protein and oil worldwide. However, its yield is strongly affected by various pests and pathogens, including plant-parasitic nematodes (PPN). The soybean cyst nematode, *Heterodera glycines* Ichinohe, a sedentary and endoparasitic nematode, is one of the most damaging pests attacking soybean (Niblack et al., 2006). An estimated annual soybean yield loss by *H. glycines* has been reported to be around US$1 billion in the United States (Koenning and Wrather, 2010). This nematode is also a major constraint to soybean production in China (Ou et al., 2008; Zheng et al., 2009; Wang et al., 2015; Peng et al., 2016). The conventional management strategies rely heavily on the combination of non-host crop rotation (e.g., maize, *Zea mays* L.) and the use of resistant host cultivars in the field. However, the limited availability of genetic resources for resistance to *H. glycines* and the genetic variation in the field population of *H. glycines* restrict the application of this method (Chen et al., 2001; Koenning, 2004; Niblack et al., 2008). In addition, the use of highly effective nematocide is prohibited in some regions because of environmental health and safety concerns (Mereu and Chapman, 2010; UNEP, 2015). Recently, some biocontrol agents were reported to reduce *H. glycines* reproduction in field conditions (Jensen et al., 2018; Lund et al., 2018; Zhao et al., 2019, 2020), but the efficacy of the biological agents may be easily affected by environmental conditions in the field. Thus, alternative new approaches are needed for controlling this widespread and destructive pest.

The neuromuscular system is considered as the potential target of the effective chemotherapeutic treatments for animal-parasitic nematode management because a series of normal parasite biological functions is governed through the nerve-muscle coordinated function (Maule et al., 2002; McVeigh et al., 2012). The role of the neuromuscular system in PPN has also been demonstrated in various facets of parasitic activities, such as host location, penetration, and migration inside and outside of host tissues, and even slight head motion at the feeding site (Perry, 1996; Maule et al., 2002; Curtis, 2008; Reynolds et al., 2011; Han et al., 2018). There is an increasing interest in understanding the potential for exploiting key regulatory genes embedded within the neuromuscular system as a target resource to develop PPN control strategies in the agricultural system. Several neuron-related genes have been identified and cloned from PPN (Yan and Davis, 2002; Costa et al., 2009; Kang et al., 2011; Shivakumara et al., 2019). For example, acetylcholinesterases (ACEs) are important in the regulation of neuronal synaptic transmission, *in vitro* RNA interference, or plant- expressing dsRNAs to effect silencing of *ace* genes significantly inhibited the invasion, development, and parasitic abilities of PPN (Jae et al., 2011; Cui et al., 2017). Most importantly, transgenic expression of the synthetic peptides, which can disrupt nerve impulse transmission dependent on ACE, conferred plant resistance to *Globodera pallida*, *Radopholus similis*, and *Meloidogyne incognita* in potato, plantain, and eggplant, respectively (Liu et al., 2005; Lilley et al., 2011; Roderick et al., 2012; Papolu et al., 2020). *In vitro* silencing of two neuropeptide genes, *Mi-nlp-3* and *Mi-nlp-12*, *via* RNA interference (RNAi) also displayed a significant reduction in attraction and penetration of *M. incognita* in tomato root in the Pluronic gel system (Dash et al., 2017). Some neuropeptide-like proteins (NLP) identified from PPN were found to negatively regulate chemosensation, host invasion, and stylet thrusting of *M. incognita* and *G. pallida*, and exogenous application of the transgenic *Bacillus subtilis* and *Chlamydomonas reinhardtii* secreting these NLP can protect tomato from *M. incognita* and *G. pallida* invasion (Warnock et al., 2017). The recently reported silencing of chemosensory genes, *Mi-odr-1*, *Mi-odr-3*, *Mi-tax-2*, and *Mi-tax-4*, which are located in the amphidial neuron and phasmid of *M. incognita*, resulted in defects in the host recognition and invasion (Shivakumara et al., 2019). In this direction, the neuromuscular system may provide attractive and alternative targets for *H. glycines* control as part of an integrated management strategy.

FMRFamide-like peptides (FLPs), with a C-terminal Arg-Phe-NH_2_ motif, are known to be the largest family of nematode neuropeptides and are widely expressed in the nervous system. Available EST/genome/transcriptome data suggest that there are at least 32 *flp* genes in model nematode species encoding >70 possible distinct peptides (McCoy et al., 2014). FLPs have been extensively studied for their core roles in the neuromuscular function of *Caenorhabditis elegans* and animal-parasitic nematodes (Li and Kim, 2014; McCoy et al., 2014; Peymen et al., 2014). Only a few *flp* genes have been successfully cloned from PPN, including *M. incognita* (Dalzell et al., 2009; Roderick et al., 2012), *Meloidogyne graminicola* (Kumari et al., 2017), *G. pallida* (Kimber et al., 2001; Dalzell et al., 2009; Atkinson et al., 2013), and *Heterodera avanae* (Thakur et al., 2012; Dutta et al., 2020). RNAi-mediated suppression of the *flp* genes in PPN resulted in the reduction of locomotion and chemosensory activities of second-stage juveniles (J2s), thereby impairing their infection ability (Kimber et al., 2007; Dalzell et al., 2009, 2010; Papolu et al., 2013; Dong et al., 2014; Kumari et al., 2017; Dutta et al., 2020). Additionally, several studies have successfully applied the method of host-derived RNAi to reduce PPN infection and reproduction by targeting *flp* genes (Papolu et al., 2013; Dutta et al., 2020; Hada et al., 2020). More recently, Banakar et al. (2020) generated transgenic tobacco using hairpin fusion expressing gene cassette of FLPs containing *Mi-flp-1*, *Mi-flp-12*, and *Mi-flp-18*, which significantly reduced the reproduction of *M. incognita*. Surprisingly, *flp-32* of *G. pallida* is the first FLP-encoding gene reportedly, which negatively regulates nematode movement by activating its putative receptor (Gp-flp-32R; Atkinson et al., 2013).

To date, studies of FLP functions in PPN have primarily focused on root-knot nematodes, *M. incognita* and *M. graminicola*; only *in vitro* physiological experiments have tested the proteolysis of two synthetic peptides (FLP-6 and FLP-14) and their effects on the behavior of *H. glycines* J2s (Masler, 2010; Masler et al., 2012). Chromatographic analysis of the FaRP immunoactivity from *H. glycines* further indicated that FLPs are present in all development stages and the highest FLP immunoactivity occurs in the J2 stage (Masler et al., 1999). Early studies also revealed the obvious immunoactivity to FMRFamide in the nervous system of *H. glycines* (Atkinson et al., 1988). These observations suggested that the *flp* family may have an important role in the behavior and parasitism of *H. glycines*. Previously, in order to understand the molecular basis of *H. glycines* recognizing host signals before J2s penetrate soybean roots and to identify new genes potentially involved in nematode chemoreception, we generated the transcriptome sequence data from *H. glycines* J2s attracted to host roots in the PF-127 gel system. Based on previous reports (McVeigh et al., 2006; Li and Kim, 2014) and our transcriptome annotation data of *H. glycines* J2s (unpublished), at least 20 *flp* genes are present in *H. glycines*. However, little is known about the functional characteristics of *flp* genes in *H. glycines*. In the present study, Hg-FLP-22 encoding gene was identified and cloned from *H. glycines* J2s, and its expression pattern was analyzed by quantitative real time (qRT)-PCR in different development stages. The functional characterization of *Hg-flp-22* was studied in behavior, infection, and development of *H. glycines via* RNAi and exogenous application of synthetic peptides.

## Materials and Methods

### Nematode Culture

*Heterodera glycines* race 5 was cultured on a susceptible commercial soybean cultivar “DongSheng1” in a greenhouse at 22–28°C in the Northeast Institute of Geography and Agroecology, Harbin, China. Cysts were collected from the soil and soybean roots from the culture pots. Eggs were released by manually crushing cysts with a rubber stopper and then harvested by pouring the solution through a 25 μm pore sieve. Collected eggs were further isolated from debris through sucrose (100%, w/v) centrifugation and thoroughly washed in distilled water. Eggs were kept in 3 mM ZnSO_4_ solution in an incubator at 28°C for hatching. Fresh J2s were collected at 3–4 days and then used immediately in each experiment. A pure culture of *M. incognita* was maintained on tomato cv. “Zhongshu-4” in the greenhouse under the same conditions as mentioned above. Nematode eggs and J2s were collected as previously described by Hu et al. (2017a).

### Isolation of *Hg-flp-22* Gene From *H. glycines*

A putative full-length cDNA encoding FLP-22, designated *Hg-flp-22*, was identified from this study of transcriptome sequence data of *H. glycines* J2s. Specific primers (Supplementary Table 1) were designated to amplify *Hg-flp-22* cDNA containing the predicted full-length open reading frame (ORF) and partial 3' untranslated region (UTR) using Phanta Super-Fidelity DNA Polymerase (Vazyme, Nanjing, China). The cycling conditions consisted of an initial denaturation at 95°C for 3 min, followed by 35 cycles of 95°C for 10 s, 60°C for 15 s, and 72°C for 30 s, followed by a final extension at 72°C for 5 min. The amplified PCR product was purified and cloned into pLB fast cloning vector (TIANGEN, Beijing, China), and then transformed into *Escherichia coli* DH5α chemically competent cells (Biomed, Beijing, China). Recombinant plasmids were isolated from at least four positive clones and sequenced by TSINGKE biological Technology (Harbin, China). Return sequences were analyzed by using BioEdit version 7.2.5.

### Bioinformatic Analysis

To perform sequence homology comparisons, Basic Local Alignment Search Tool (BLAST) searches were conducted in the nematode non-redundant and expressed sequence tag (EST) databases at the National Centre for Biotechnology Information (NCBI) BLAST server by using BLASTX and BLASTN. The predicted protein sequences of *Hg-flp-22* and its homologs across the nematode species were aligned using online multiple sequence alignment tools with default settings.^1^ A phylogenetic tree was constructed using MEGA6 software with the neighbor-joining algorithm based on the Poisson distance correction, and the bootstrap test was performed with 1,000 replicates.

### Peptide Synthesis and Treatment

Peptides QPAGGVKWMRFa (Hg-FLP-22a), TPQGKWMRFa (Hg-FLP-22b), and KMAIEGGKWVRFa (Hg-FLP-22c) were synthesized by SangonBiotech (Shanghai, China) at 95% purity. Each synthesized peptide was dissolved to sterilized ddH_2_O to make a 10 mM stock solution and was then aliquoted and stored at −20°C. For nematode behavioral assay, J2s of *H. glycines* or *M. incognita* were incubated for 15 min in 10 μM, 100 μM, or 1 mM solution of peptides in a PCR tube. For infection assay, J2s were added to 400 μl of peptides solution and incubated for 16 h at room temperature in the darkness. All of the peptides stock stored at −20°C were used within 1 week.

### *In vitro* RNAi of *Hg-flp-22* in *H. glycines* J2s

Double stranded RNA (dsRNA) of *Hg-flp-22* (250 bp) were synthesized and purified with a MEGAscript® RNAi Kit (Thermo Fisher Scientific, Waltham, MA, United States) according to theinstructions of the manufacturer using the primers with the T7 promoter sequence appended to the 5' end (Supplementary Table 1). dsRNA of an unrelated gene *gfp* (720 bp) was synthesized from a plasmid containing *gfp* fragment and used as a non-native negative control. dsRNA soaking of J2s was performed as previously described by Urwin et al. (2002). Approximately 4,000 freshly hatched J2s were soaked in the soaking buffer containing 2 mg/ml dsRNA, 3 mM spermidine, and 50 mM octopamine at room temperature on a rotator in the dark. J2s incubated in dsRNA of *gfp* gene and soaking buffer (without dsRNA) were set as the control. After 24 h incubation, J2s were washed four times with sterile distilled water through centrifugation to remove the external dsRNA. Additionally, dsRNA uptake efficiency was assessed by using fluorescein isothiocyanate (FITC, 0.1 mg/ml, Sigma, St. Louis, MO, USA) as a tracer in dsRNA buffer, and then, nematodes were observed under a fluorescence microscope (LEICA DM2500, Wetzlar, Germany).

Approximately 500 dsRNA-treated J2s were used for total RNA isolation and qRT-PCR analysis of the transcribed abundance of the target gene. The remaining J2s were used to assess behavior, infection, and development of nematode.

### Analysis of *H. glycines* J2 Behavior

For head movement assay of nematode, approximately 100 J2s were incubated in peptides solution for 15 min. Then, J2s were transferred to a cell counting chamber (75 mm × 25 mm × 1.8 mm; chamber depth = 100 μm) with four rooms (10 μl loading volume), and head movements were observed for a randomly selected individual nematode under a stereomicroscope (OLYMPUS SZX-16, Tokyo, Japan). Head movement frequencies of J2s were determined following a definition previously described by Masler et al. (2012). Head movements of each J2 were counted for 1 min. At least 60 J2s from three independent experiments were counted for each peptide treatment.

In the present study, root extracts from 8-day-old soybean plants were used as a source of an attractant to test the chemotaxis effect. Briefly, 0.5 g fresh root tips (0.5 cm) were homogenized in 4 ml sterile distilled water, and homogenates were centrifuged at 12,000 *g* for 10 min at 4°C. The supernatant was then collected, filter sterilized (0.22 μm, Merck Millipore, Billerica, MA, USA), and used for chemotaxis assay (Hu et al., 2019). Fresh root extracts were collected from each independent experiment. Chemotaxis assays were performed in a 60 mm Petri dish according to the methods previously described (Hu et al., 2019). The dish was filled with 5 ml 1% agar (DAISHIN, Dublin, OH, USA) and allowed to solidify. Two points (A and B) were marked on the back of the dish 2 cm on either side of the dish center. Root extracts of 5 μl were applied at the A point and 5 μl of sterile distilled water (as a control) was added at the B point. After 10 min, approximately 100 J2s were added to the center of the plate, which is also the midpoint of the two marked points. The Petri dishes were placed at room temperature for 4 h in an incubator, and the number of J2s in the vicinity of the injection site of extracts or control was counted. Chemotaxis index was calculated as the number of J2s near the A point minus the number of J2s near the control B point divided by the total number of J2s present at A and B. The experiment was carried out two times with eight replicates each time.

*Heterodera glycines* attraction assays were conducted in six-well tissue culture plates containing Pluronic F-127 gel (NF Prill Poloxamer 407, BASF, Mount Olive, NJ, United States; Hu et al., 2017b). About 3 ml of 23% (w/v) Pluronic F-127 was added into each well at 4°C, and a 1 cm root piece (with an intact tip) of 4-day-old soybean seedling was placed in the gel. The culture plates were then transferred to room temperature conditions. After solidification, approximately 200 dsRNA-treated J2s were added into each well, and the injection site was about 1 cm from the root tip. The attractiveness of soybean roots to *H. glycines* was observed using a dissecting microscope, and the number of J2s touching the root surface or within 5 mm of the root tip was counted at 4 h. In addition, the root pieces were collected from gel at 7 and 16 h after starting the assay, and the number of J2s which had penetrated the root was determined by root staining with acid fuchsin (Byrd et al., 1983).

### Nematode Infection in Soil

Soybean seeds were surface-sterilized in 1.5% sodium hypochlorite for 15 min, rinsed thoroughly with tap water, and germinated for 4 days in darkness at 26°C, after which they were transferred to pots containing sterilized sand and soil (2:1) and grown in a growth chamber with a photoperiod of 16 h light (26°C) and 8 h dark (20°C) cycle. After 8 days, soybean seedlings were inoculated with 200 *H. glycines* J2s per plant, the J2s have been pretreated with peptides or dsRNA. All plants were placed in a completely randomized design in a growth chamber and eight biological replicates of each treatment per time-point were used. At 1 day after the nematode inoculation (dai), root systems were harvested and the penetration ability of the nematodes was determined under a stereoscope through acid fuchsin staining. The number of females on the roots was counted under a stereomicroscope using a counter at 21 dai.

For *M. incognita* inoculation, a similar procedure was used but with a tomato host plant. About 200 J2s were applied on each 2-week-old tomato seedling (cv. Zhongshu-4) which was grown and maintained in a sterilized soil mixture (1:1 mixture of sand and soil) in a 10 cm × 9.5 cm tray, and then, the number of nematodes that had penetrated the host roots was counted at 1 dai. During the course of the experiment, plants were watered four times a week, with one of the watering consisting of Hoagland’s nutrient solution (Hoagland and Arnon, 1950). The experiment was conducted three times with at least eight replicates for each time.

### RNA Extraction, cDNA Synthesis and qRT-PCR Analysis

For development expression level assay, individuals (1,000–2,000) in different life stages of *H. glycines* (including eggs, pre-parasitic J2s, parasitic J2s, mixed J3s/J4s, adult females, and males) were used for RNA extraction. The infected roots were harvested at 3 and 12 dai, and the parasitic J2s and mixed J3s/J4s were extracted according to the method as described by Wang et al. (2014). Briefly, the roots were homogenized for 20 s in a juice extractor, and the homogenate was washed through successive 850, 90, and 25 μm pore sieves. Then, the filtrate containing nematodes was purified by a sucrose (50%, w/v) gradient. Males and adult females were obtained from soybean roots at 16 and 21 dai, respectively, in the pouch system (You et al., 2018). Total RNA of nematode samples was purified using an RNAPrep Pure Micro Kit (TianGen Biotech, Beijing, China) following the instructions of the manufacturer including a step DNase treatment. First-strand cDNA was synthesized from 0.5 μg of total RNA using FastKing gDNA Dispelling RT SuperMix FastKing Kit (TianGen Biotech, Beijing, China). qRT-PCR analysis was carried out in the LightCycler® 480 System with AceQ qPCR SYBR Green Master Mix (Vazyme, Nanjing, China) according to the procedure described by the manufacturer. Cycle conditions were 95°C for 5 min and next 40 cycles of 10 s at 95°C and 30 s at 60°C. The 2^−∆∆Ct^ method was applied to calculate the related expression of *Hg-flp-22* in different life stages and dsRNA treatments (Hu et al., 2017b). *Hg-GAPDH* was used as a reference control to normalize all qRT-PCR results (Wang et al., 2014). All experiments were performed with three independent biological replicates, each with three technical replicates. The primers used for qRT-PCR were listed in Supplementary Table 1.

### Data Analysis

Data were subjected to one-way ANOVA using SPSS version 17.0 software (Chicago, IL, United States). Tukey’s HSD test (*p* < 0.05) was used for overall pairwise comparisons. The error bars in the figures represent the SD of means, and the significance level was set at *p* < 0.05.

## Results

### Identification and Sequence Analysis of *Hg-flp-22* From *H. glycines*

**Figure 1:**
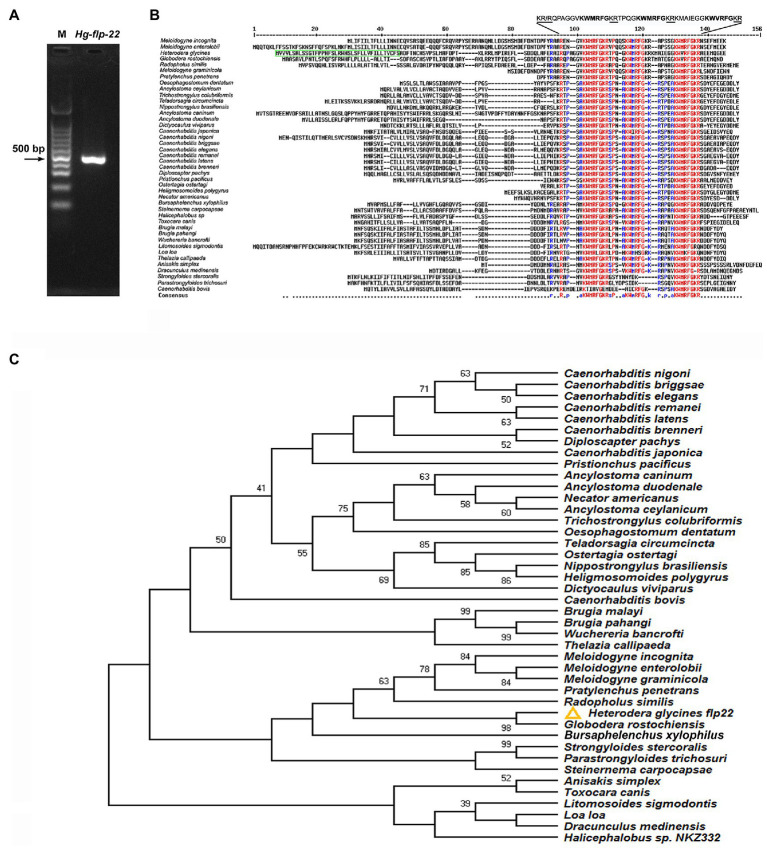
**(A)** PCR amplification of *flp-22* cDNA fragment of *Heterodera glycines*. M: 100 bp marker. **(B)** Multiple sequence alignment of FLP-22 peptide from *H. glycines* (GenBank Accession No. MW645239) with homologs from other nematode species using MULTALIN online tool (http://multalin.toulouse.inra.fr/multalin/). The predicted signal peptide of Hg-FLP-22 is marked by a green box. Residues identical to those of FLP-22 are indicated by the identical color of amino acids. Conserved Hg-FLP-22-like motifs are flanked by mono (R) or dibasic (KR) cleavage sites. **(C)** Phylogenetic tree of nematode *flp-22* genes. Putative FLP-22 peptides from *H. glycines* and other 41 nematode species were obtained with BLAST using *Hg-flp-22* sequence as a query. The evolutionary tree was generated by using the Neighbor-Joining method based on Poisson distance correction. The bootstrap analysis consisted of 1,000 replicates, and the cutoff value for a condensed tree was 38%.

A putative *flp-22* gene of *H. glycines* was identified based on our transcriptome sequence data of *H. glycines* J2s. The cDNA fragment of *Hg-flp-22* (479 bp) containing the predicted full-length ORF and partial 3' UTR was amplified using specific primers (Figure 1A; Supplementary Table 1). The ORF (GenBank Accession MW645239) encodes a protein precursor of 129 amino acids, including a secretion signal peptide of 38 amino acids at its N-terminal (Figure 1B). BLAST analysis was conducted in the available nematode EST, genomic, and transcriptomic databases using *Hg-flp-22* as a search query, and *Hg-flp-22* complements were identified in 42 nematode species, including free-living and animal- and plant-parasitic (Supplementary Table 2). Multiple sequence alignment results showed a considerable sequence diversity of FLP-22 among these species (Figure 1B). In free-living nematodes, *Caenorhabditis* spp., except *C. bovis*, *flp-22* gene encodes a single peptide SPSAKWMRF-NH_2_ with three copies, while *flp-22* genes from other nematodes are translated to three distinct active peptides with a common C-terminal KWMRF-NH_2_ and a poorly conserved N-terminal. It is worth noting that Hg-FLP-22 was more similar to a homolog from *Globodera rostochiensis* (Gr-FLP-22, BM343164.1), sharing 72% sequence identity; indeed, the identity of two peptides, QPAGGVKWMRFa and TPQGKWMRFa, in *H. glycines* and *G. rostochiensis* were 100% (Figure 1B). Phylogenetic analysis showed that *flp-22* of *H. glycines* was clustered with the *flp-22* complements of PPN and animal-parasitic nematodes, *Strongyloides stercoralis*, *Strongyloides ratti*, *Parastrongyloides trichosuri*, and *Steinernema carpocapsae* (Figure 1C).

### Expression of *Hg-flp-22* in Different Development Stages of *H. glycines*

**Figure 2:**
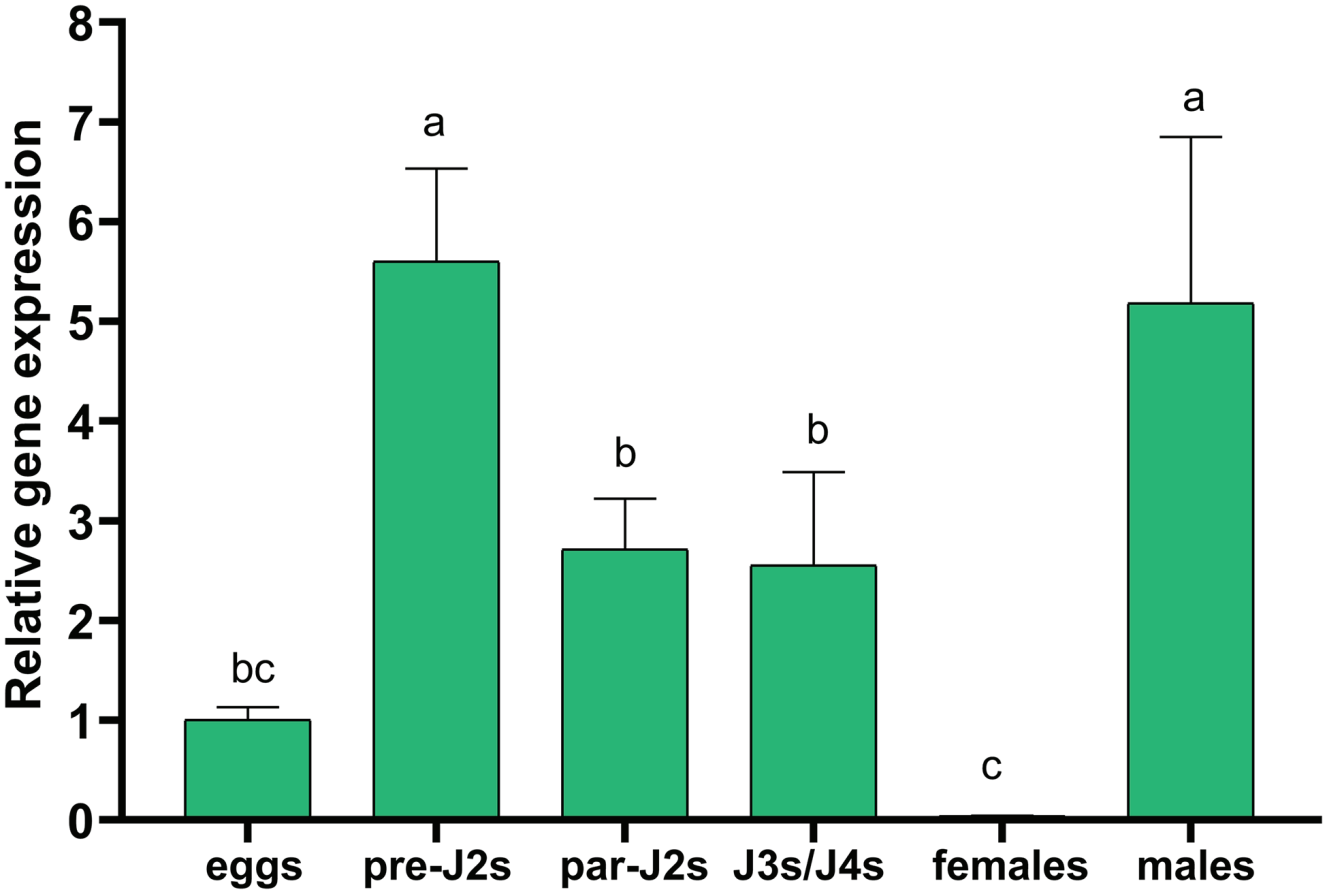
The expression levels of *Hg-flp-22* by quantitative real-time (qRT)-PCR analysis in eggs, pre-parasitic J2s (pre-J2s), parasitic J2s (par-J2s), J3s/J4s, and adult females and males. Gene expressions were calculated by using the 2^−ΔΔCT^ method and were presented relative to those of eggs (normalized with an endogenous reference gene *Hg-GAPDH*). Values are means ± SD from three biological replicates. Bars with the same letters indicate no significant difference at *p* > 0.05 (One-way AVOVA, Tukey’s HSD test).

The transcript levels of *Hg-flp-22* were analyzed at six different parasitic-stages (eggs, pre-parasitic J2s, parasitic J2s, J3s/J4s, and adult females and males) using the expression levels in eggs as a reference by qRT-PCR. *Hg-flp-22* was expressed at the highest levels in pre-parasitic J2s and adult males compared with other life stages. Although its transcript levels declined during the parasitic J2 and J3/J4 stages, it also exhibited, respectively, approximately 2.7- and 2.6-fold higher transcripts than those of the eggs. The lowest expression levels of *Hg-flp-22* were found in adult females in contrast to its upregulation in early parasitic-stages of *H. glycines* (Figure 2). We tried to use *in situ* hybridization assay to detect the tissue localization of *Hg-flp-22* in the neuron system of pre-parasitic J2s, but DIG-labeled probes specific to *Hg-flp-22* did not exhibit specific neural or non-neural tissue expression pattern even though a detectable signal was found in the anterior and tail regions of J2s (Supplementary Figure 1).

### *In vitro* RNAi of *Hg-flp-22* Affects Infection and Development of *H. glycines*

**Figure 3:**
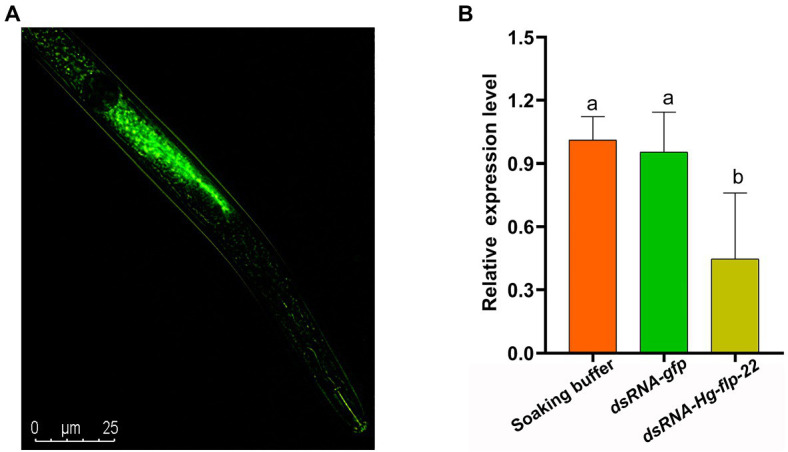
**(A)** Fluorescence microscopy indicating uptake of fluorescein isothiocyanate (FITC) in dsRNA buffer by *H. glycines* J2s after 24 h incubation. **(B)** Pre-parasitic J2s were incubated in the socking buffer, *Hg-flp-22* dsRNA, or *gfp* dsRNA for 24 h. Then, the *Hg-flp-22* transcript levels in nematodes were analyzed by qRT-PCR. The results are the means ± SD of three biological replicates shown relative to expression in J2s maintained in the socking buffer. The same letters indicate no significant differences between treatments (*p* > 0.05, Tukey’s HSD test).

**Figure 4:**
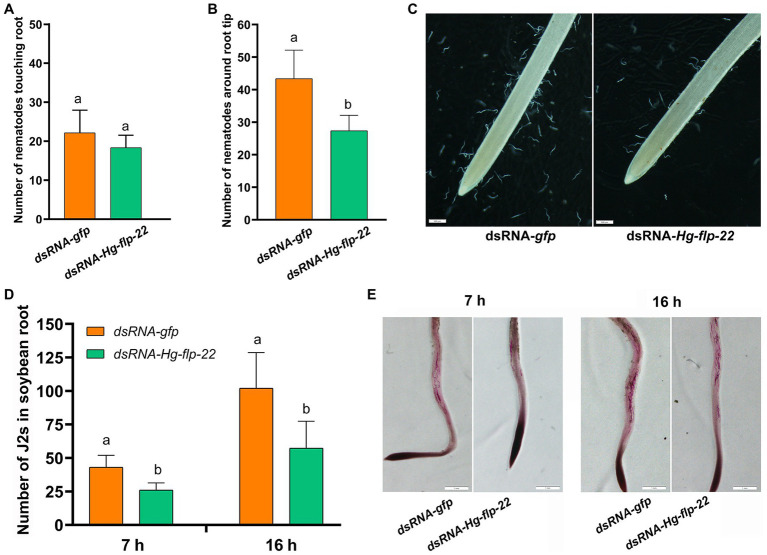
Effect of *in vitro* RNAi of *Hg-flp-22* on *H. glycines* attraction and penetration in PF-127 medium. **(A)** The number of nematodes touching or within 5 mm of the root was counted at 4 h after starting the assay. Data are the means ± SD (*n* = 10) and are representative of three independent experiments. Statistical analysis was performed using Tukey’s HSD test. The same letters indicate no significant differences between treatments (*p* > 0.05). **(B)** The number of nematodes around the root within the terminal 5 mm was counted at 4 h after starting the assay. Data are the means ± SD (*n* = 10), and bars with the same letter indicate no significant difference at *p* > 0.05 (Tukey’s HSD test). J2s soaked with *gfp* dsRNA were used as the control. **(C)** Representative images showing the attraction of *Hg-flp-22* dsRNA-treated J2s toward soybean root tips in PF-127 medium at 4 h after starting the assay. Scale bar = 500 μm. **(D)** The number of stained J2s inside the root was counted at 7 and 16 h after starting the assay. Values are the mean ± SD from one representative experiment (*n* = 10). The same letter indicates no significant differences at *p* > 0.05 (Tukey’s HSD test). J2s treated with *gfp* dsRNA were used as the control. **(E)** Stained J2s in soybean roots. Scale bar = 1 mm.

To study the role of *Hg-flp-22* in *H. glycines* parasitism, the universal RNAi technology *in vitro* was used to investigate whether silencing of *Hg-flp-22* expression can affect mobility, host recognition, penetration, and development of *H. glycines*. Fluorescence microscopy indicated that dsRNA was efficiently absorbed into J2 bodies (Figure 3A). qRT-PCR analysis of efficiency for gene-silencing showed that the transcript levels of *Hg-flp-22* were significantly decreased by 54% in *H. glycines* J2s after soaking in target dsRNA (2 mg/ml) when compared with those in the soaking buffer without dsRNA. Non-native control dsRNA-*gfp* treatment did not result in a statistically significant reduction in the target transcript of *Hg-flp-22* (Figure 3B). The host-searching behavior of J2s toward soybean root tips, which requires a combination of abilities of nematode locomotion and chemosensation, was assessed in Pluronic F-127 (PF-127) gel system (Hu et al., 2017b). Normally, infective J2s of *H. glycines* can recognize the presence of signals from the host root exudates and are preferentially attracted to soybean root tips, especially the elongation zone (Figure 4). Although, no significant difference was observed in the number of nematodes touching the root between the target gene dsRNA-treated group and the *gfp* dsRNA-treated control 4 h post-exposure (Figure 4A). Silencing of *Hg-flp-22* resulted in lower J2 attraction to the vicinity of the soybean root tip (Figures 4B,C). The average number of J2s treated with *Hg-flp-22* dsRNA surrounding the root tip was 43.4 ± 8.8, whereas it was 27.4 ± 4.7 for *gfp* dsRNA-treated nematodes (Figures 4B,C). Once they reach the host root tips, J2s of *H. glycines* use their stylet to invade the host root within 2 h after the assay started in PF-127 medium (Hu et al., 2017b). Therefore, the effect of *in vitro* RNAi of *Hg-flp-22* on the penetration ability of J2s was further evaluated at 7 and 16 h post-exposure. Compared to the *gfp* dsRNA-socking nematodes, a significantly lower number of target dsRNA-treated J2s were observed to penetrate the host root at 7 and 16 h (Figures 4D,E).

**Figure 5:**
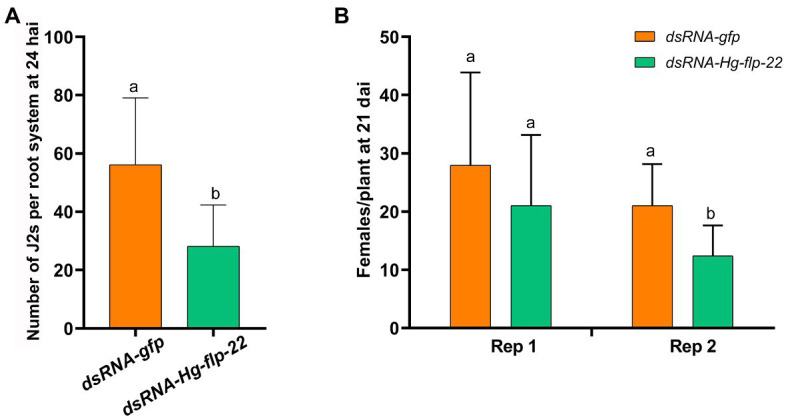
Effect of *in vitro* RNAi of *Hg-flp-22* on *H. glycines* infection and development in soil. **(A)** The penetration ability of *Hg-flp-22*-silenced or *gfp* dsRNA-treated nematodes in soybean roots grown in pots containing sterilized sand and soil. Each plant was inoculated with 200 J2s. The number of nematodes in soybean roots was counted at 24 h after inoculation (hai) through acid fuchsin staining. Data are the means ± SD from eight plants and are representative of two independent experiments. The same letter denotes no significantly difference in the number of *Hg-flp-22* dsRNA-treated J2s (*p* > 0.05, Tukey’s HSD test) compared with that of nematodes soaked with *gfp* dsRNA. **(B)** Females in soybean roots were observed 21 days after inoculation (dai) in two independent experiments. Values are the means ± SD from eight plants. Bars with the same letter indicate no significant difference at *p* > 0.05 (Tukey’s HSD test). J2s treated with *gfp* dsRNA were used as the control.

Because *H. glycines* are soil-borne in nature, we further compared the infection and development of the host root by the *Hg-flp-22* dsRNA-treated J2s to the *gfp* dsRNA-treated nematodes in soil assay. Results showed that silencing of *Hg-flp-22* decreased the number of the stained J2s inside roots by 49.9% at 24 h post inoculation (hpi; Figure 5A). A significant reduction in the number of adult females on the root system at 21 days after the nematode inoculation (dpi) of target dsRNA-treated J2s was found in one independent biological experiment assay compared with that of the *gfp* dsRNA-treated J2s (Figure 5B).

### Exogenous Application of Synthetic Peptides Affects the Behavior of *H. glycines*

**Figure 6:**
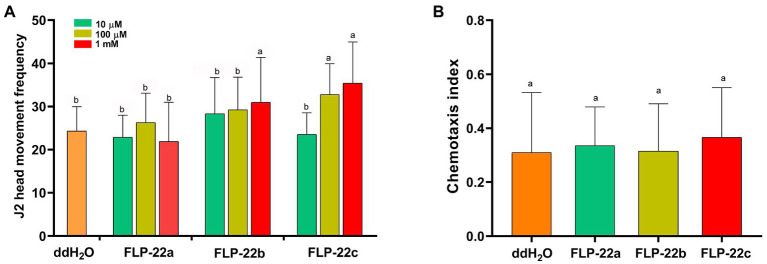
Effect of three mature Hg-FLP-22 peptides on the *in vitro* behavior of *H. glycines* J2s. **(A)** Effect of Hg-FLP-22 peptides on the frequency of J2 head movements per minute. Each value represented the mean head movement frequency ± SD from 60 individual nematodes. The same letters indicate no significant differences between the peptide treatments and ddH_2_O control (*p* > 0.05, Dunnett’s test). **(B)** Effect of Hg-FLP-22 peptide treatments on chemotaxis indices of *H. glycines* J2s. Each bar represents the mean ± SD of the data from the two independent experiments. The same letters indicate no significant differences from ddH_2_O control (*p* > 0.05, Dunnett’s test).

**Figure 7:**
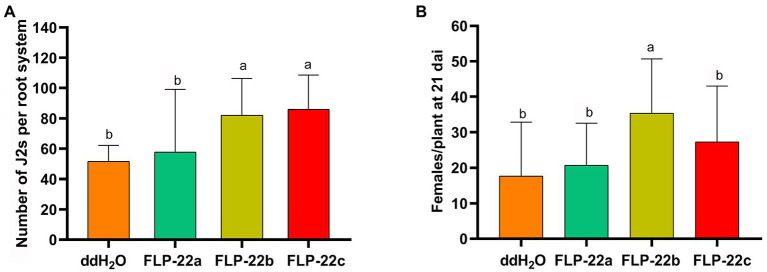
Effect of Hg-FLP-22 peptides on infection and development of *H. glycines* in soil. **(A)** Host invasion ability of Hg-FLP-22 peptides or ddH_2_O-treated nematodes in soybean roots in pots containing sterilized sand and soil. Each plant was inoculated with 200 J2s. The number of nematodes in soybean roots was counted at 24 hai through acid fuchsin staining. Bars are the mean ± SD from one representative experiment (*n* = 8). These experiments were repeated three times with similar results. **(B)** Females in soybean roots were counted 21 dai. Values are the means ± SD from eight plants and are representative of three independent experiments. The same letters indicate no significant differences between the peptide treatments and ddH_2_O control as determined by Dunnett’s test at *p* > 0.05.

We, next, determined whether synthetic Hg-FLP-22 peptide treatments could produce the opposite phenotype of *Hg-flp-22*-silenced nematodes. *H. glycines* J2s were incubated in a series of concentrations of the synthetic Hg-FLP-22 peptides for 15 min, and the effects of peptide treatments on head movement frequency in cycles per minute of individual nematodes were assessed. Treatment with high concentrations of FLP-22b (1 mM) led to approximately 27.3% increase in the mean head movement frequency compared with the ddH_2_O-treated nematodes. Significant stimulatory effects of FLP-22c on the head movement of *H. glycines* were found at both 10 μM and 1 mM peptide solutions. In contrast, FLP-22a had no significant effect on head movement frequency (Figure 6A). Chemotaxis assay suggested that none of the synthetic Hg-FLP-22 peptides treatments can affect the normal chemotaxis toward soybean root exudate (Figure 6B). Infection assay indicated that many more FLP-22b- (82 ± 12, *p* < 0.05) and FLP-22c-treated (86 ± 12, *p* < 0.05) J2s penetrated into soybean roots compared with the ddH_2_O-treated control nematodes (51 ± 12, *p* < 0.05; Figure 7A). FLP-22b treatment also caused a significant increase in the number of females on the host roots relative to the ddH_2_O-treated control at 21 dpi (Figure 7B).

In PPN, *flp-22* gene encodes three mature peptides with the most highly conserved C-terminal motif (-GVKWMRFG, -Q**G**/**S**KWMRFG, and -GKWM/VRFG; Supplementary Figure 2A). We, therefore, investigated whether the other PPN have similar behavioral responses to the synthetic Hg-FLP-22 peptides. The same head movement and infection assays were performed in *M. incognita* J2s exposure to 1 mM FLP-22a, FLP-22b, or FLP-22c. When compared to *H. glycines* J2s, three synthetic Hg-FLP-22 peptides failed to significantly stimulate the head movement of *M. incognita* J2s, and only FLP-22b displayed a slight stimulatory effect on the frequency of head movement (Supplementary Figure 2B). Additionally, the number of synthetic peptides-treated and ddH_2_O-treated J2s of *M. incognita* in tomato roots was not significantly different (Supplementary Figure 2C).

## Discussion

Increasing evidence for FLP-mediated biological functions in host finding, invasion, and reproduction of PPN support the potential valuable repository of FLP genes as possible targets for the management of plant pathogenic nematodes (Kimber et al., 2007; Dalzell et al., 2010; Papolu et al., 2013; Dong et al., 2014; Kumari et al., 2017; Banakar et al., 2020). Previous interrogation of *H. glycines* EST database by McVeigh et al. (2006) identified two *flp-22* EST transcripts. In this study, FLP-22 encoding gene *Hg-flp-22* in *H. glycines* was successfully cloned and characterized in detail. Bioinformatic analysis revealed the high degree of conservation of the amino acid sequences of *Hg-flp-22* and homologous genes from other nematode species, especially a characteristic C-terminal KWMRF-NH_2_ motif, suggesting that *flp-22* may have the conserved physiological function in phylum Nematoda.

Quantitative real time-PCR analysis demonstrated that *Hg-flp-22* is predominantly expressed in pre-parasitic J2s and adult males but that it showed a significantly reduced expression in adult females compared with other stages. This expression profile may be associated with the remodeling of the neuromuscular structure during the mobile stage of *H. glycines* transition to immobility and the resumption of mobility in males (Han et al., 2018). Similar expression profiles in mobile J2s and adult males were also reported in other *flp* genes in *M. graminicola* (*flp-1*, *flp-3*, *flp-6*, *flp-7*, *flp-11*, *flp-12*, *flp-14*, *flp-16*, and *flp-18*; Kumari et al., 2017) and *M. incognita* (*flp-14* and *flp-18*; Papolu et al., 2013). The degeneration of specific neuromuscular cells may lead to the lower transcript levels of these *flp* genes in the adult female stage of PPN (Bird, 1967; Han et al., 2018). These observations suggest the important role of the *flp* family in the early parasitic process and the male mating behavior of PPN. Before the onset of the sedentary stage, mobile parasitic-J2s travel toward the vascular cylinder of host roots and initiate the formation of a specialized feeding site, from which nematode withdraws nutrients throughout its life (Davis et al., 2004). All of these continuous actions, such as stylet movement, effector secretion from an esophageal gland, and swallowing nutrients, are dependent on the function of motor neurons. Despite the emergence of cell-specific body muscle atrophy in *H. glycines* during the sedentary stages, the atrophy in head and esophageal muscles is not observed in the immobile nematodes, suggesting that the persistent head movement and stylet thrusting are also essential for *H. glycines* feeding behavior from syncytium (Han et al., 2018). The data showed that *Hg-flp-22* displayed relatively high transcripts in parasitic-J2 and J3/J4 stages, together with the expression pattern of *flp-22* in many cell-types, such as muscle cells, as well as the physiological phenotypes of FLP-22 peptide-treated nematodes (Moffett et al., 2003; Kim and Li, 2004; Papaioannou et al., 2005), and it is possible that *Hg-flp-22* may also exert its functions on the development of the sedentary *H. glycines*.

A remarkable abundance of *flp* genes is expressed in the nervous system of all nematodes, including motor neurons, sensory neurons, and interneurons (Li and Kim, 2014; Peymen et al., 2014). The wide distribution pattern of the *flp* genes family endows them the functional flexibility in multiple behaviors, as well as supporting muscle and somatic cells. However, the localization of *Hg-flp-22* did not display the specific expression sites of the neurons in the anterior and tail regions of *H. glycines* J2s except for a diffuse ISH staining pattern. The expression levels of *Hg-flp-22* are below the detectable threshold using the DIG-labeled probe in the present study. However, a previous study reported by Kim and Li (2004) found that the expression pattern of *flp-22* in *C. elegans* using the GFP reporter construct is widely distributed in the whole body. Most importantly, the tissue-specific location of *flp-22* transcripts of *C. elegans* was observed in multiple neuronal cell types (AIM, ASG, AVA, AVG, AVL, CEP, PVD, PVW, RIC, AIZ, RIV, and SMD) and non-neuronal cells (URA and uv1) and was mainly focused on the anterior region. Notably, published data indicated that exogenous FLP-22 peptide of *C. elegans* has markedly excitatory effect on the pharyngeal action (Papaioannou et al., 2005); other evidence suggested that FLP-22 can cause muscle contraction in the ovijector of *Ascaris suum* (Moffett et al., 2003). In *C. elegans*, body movement is dependent on muscle control, which is innervated by a series of motor neurons in the ventral nerve cord (VNC), CNR, and other nerve structures (White et al., 1986; Francis and Waterston, 1991). Considering that the neural connectivity in *H. glycines* J2s is similar to *C. elegans* (Gendrel et al., 2016; Han et al., 2018) and that Hg-FLP-22 peptides displayed the obvious stimulatory effects on the anterior movement of *H. glycines*, the distribution of *Hg-flp-22* transcript in J2 body may be involved in regulating muscle contraction and head movement of *H. glycines*.

Applications of synthetic Hg-FLP-22 peptides (FLP-22b and FLP-22c) significantly increased the head movement frequency of *H. glycines*. This is similar to the observation which showed that FLP-6 and FLP-14, both highly conserved single-copy peptides in nematode species (Peymen et al., 2014), also have significant stimulatory effects on the anterior movement frequency of *H. glycines* and *M. incognita* (Masler et al., 2012). Once hatched from eggs, the infective J2s of PPN need to quickly move toward host roots and penetrate the cortex layer cells *via* thrusting their stylet before their energy reserves are depleted (Perry, 1996). Therefore, we summarize that the high head movement frequency of pre-parasitic J2s in the soil is in favor of body motility and root penetration. Nematode infection assay indicated that *H. glycines* J2s treated with FLP-22b or FLP-22c are more quickly attracted to soybean roots and exhibited higher infection rates compared to the control treatments; this information supports the above hypothesis. Conversely, our results confirmed that loss of *Hg-flp-22* by *in vitro* RNAi decreased the J2s movement to root tips and the penetration activity in PF-127 medium and soil assay. Similar inhibitory effects on the locomotion and invasion behaviors were found in *flp-6* and *flp-14* dsRNA-treated J2s of the other PPN (Kimber et al., 2007; Papolu et al., 2013; Kumari et al., 2017), suggesting that *Hg-flp-22* may possess similar actions on neuromuscular control of *H. glycines* motor functions associated with movement and host invasion.

Lower infection ability of the RNAi-treated J2s may lead to a lower proportion of nematodes at the mature female stage. We found that silencing of *Hg-flp-22* reduced the number of females in only one independent assay, indicating that the efficacy of the RNAi experiment may be transient. Although Hg-FLP-22b treatment increased the number of females, it is difficult to interpret this information as indicative of *Hg-flp-22* function in parasitic stages because of the transient efficacy and short durability of RNAi and synthetic peptides, which have been reported in several studies (Kimber et al., 2007; Roderick et al., 2012; Kumari et al., 2017). Unfortunately, host-induced RNAi of *Hg-flp-22* was not effective in expressing *Hg-flp-22*-RNAi constructs of soybean hairy roots (data not shown), and further functional studies of *Hg-flp-22* during *H. glycines* parasitism are needed.

FMRFamide-like peptides are thought to exert most of their basic functions through the activation of cognate-receptors, and several FLP receptors have been identified in *C. elegan* by using heterologous cellular systems and genetic experiments (Li and Kim, 2014; McCoy et al., 2014; Peymen et al., 2014). In this study, the corresponding phenotypes of *H. glycines* J2s exposed to Hg-FLP-22 peptide imply that exogenous FLP peptide can be transported to neural or non-neural cells of nematode, where they can activate their cognate receptors and ultimately form the neuronal circuitry. However, the behavior potency of *H. glycines* responses to three peptides encoded on the same gene *Hg-flp-22* shows subtle differences. Hg-FLP-22b and Hg-FLP-22c displayed significant stimulatory effects on head movement and stylet penetration ability compared with Hg-FLP-22a, suggesting the two peptides may be significantly more active isoforms of Hg-FLP-22 for activation of putative cognate receptors. Similar observations were also reported by previous studies indicating obvious differences in physiological function and potency between FLP peptides encoded on the same gene in *H. glycines*, *Panagrellus redivivus*, or *C. elegans* (Masler et al., 2012; Atkinson et al., 2016). It has been documented that a single FLP receptor can be activated by multiple FLPs, whereas the potency of receptor activation by structurally similar FLPs is different (Rogers et al., 2003; Mertens et al., 2005; Cohen et al., 2009). Thus, the sequence variation of Hg-FLP-22 peptides at the N-terminal may affect ligands binding with receptor and potency, eventually resulting in the differential regulation of nematode behavior. Notably, Hg-FLP-22b and Hg-FLP-22c did not produce a significant impact on head movement frequency and infection behaviors of *M. incognita*. These results indicated that the sensitivity of nematodes to exogenous FLP-22 peptides treatment appears to be species-specific. Indeed, sequence dissimilarity of N-terminal residues is present between three FLP-22 peptides from *H. glycines* and *M. incognita*. This further supports our speculation that the conservation of the N-terminal amino acid of FLP-22 seems to be most essential for receptor activation. Another possible explanation is the difference in the metabolic levels of three peptides *in vivo*. Masler et al. (2012) reported that the *in vitro* digestion rates of FLP-14a and FLP-14b encoded by *flp-14* are different for the nematode proteolytic extraction from the same species. In fact, the posttranslational amidation of the FLP at the C-terminal is required for preventing their immediate degradation and biological function (Bowman et al., 1996). Hg-FLP-22 peptides used in the present study are in their unamidated forms and need further amidation in neuron cells following their uptake. Therefore, the lower Hg-FLP-22a amidation could result in a shorter half-life *in vivo*, which in turn leads to the low potential for regulating physiological function.

Identification of FLP-receptor couples is very important for functional studies on FLPs in nematodes. Until now, FLPs that functionally activate cognate receptors have not been reported in PPN. However, Atkinson et al. (2016) presumed that Gp-flp-32R is the cognate receptor of *Gp-flp-32* based on their matching RNAi phenotypes and expression pattern, which may not fully reflect the characterization of the FLP-GPCR relationship; more detailed receptor pharmacology experiments are necessary to further verify the observations. The Y59H11AL.1 receptor can be activated by several *C. elegans* FLP peptides including FLP-22; however, among those peptides, FLP-7 is the most active peptide (Mertens et al., 2005). In addition, knockdown of the Y59H11AL.1 receptor did not result in any defect phenotypes, such as locomotion (Keating et al., 2003). These studies suggested that Y59H11AL.1 may not be a putative receptor of FLP-22. Therefore, identification of Hg-FLP-22 receptors by using bioinformatics and receptor pharmacology experiments is required to exploit its functional characterization, as this would boost the utility of FLP-22-mediated signaling as a target for *H. glycines* control.

In conclusion, this is the first functional characterization of FLP encoding genes in *H. glycines* (*via* RNAi). These data indicate a positive regulation of bioactive FLP-22 peptides in *H. glycines* behaviors in terms of increased body movement, penetration, and development. This study also deepens the basic understanding of FLP signaling in the early parasitism of *H. glycines*, which is the basis for the exploitation of the FLP system as putative control targets.

## Data Availability Statement

The datasets presented in this study can be found in online repositories. The names of the repository/repositories and accession number(s) can be found in the article/Supplementary Material.

## Author Contributions

YH conceived and designed the experiments and wrote the manuscript. JY, FP, SW, and YW performed the experiments. JY, FP, and YH analyzed the data. All authors contributed to the article and approved the submitted version.

### Conflict of Interest

The authors declare that the research was conducted in the absence of any commercial or financial relationships that could be construed as a potential conflict of interest.

## Footnotes

^1^http://multalin.toulouse.inra.fr/multalin/
