# Supplementary material for: FMRFamide-Like Peptide 22 Influences the Head Movement, Host Finding, and Infection of Heterodera glycines
Source: Front Plant Sci. 2021 Jun 22;12:673354. doi: 10.3389/fpls.2021.673354 (PMC8258376; doi:10.3389/fpls.2021.673354)
Supplement: Supplementary file 1 [file Table_1.DOCX]

Supplementary Material

# Supplementary Figures and Tables

## Supplementary Figures


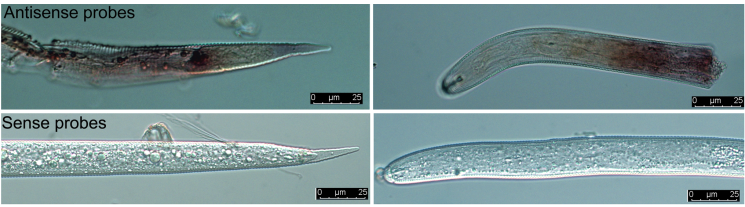


**Supplementary Figure 1.** *In situ* hybridization of DIG-labeled *flp-22* probes in the anterior and tail regions of *H. glycines* J2. DIG-labeled antisense probes specific to *Hg-flp-22* were diffusedly located in the circumpharygeal nerve ring, dorsal pharyngeal nerves, and phasmidial region of tail. No ISH staining was observed with sense probes as negative control.


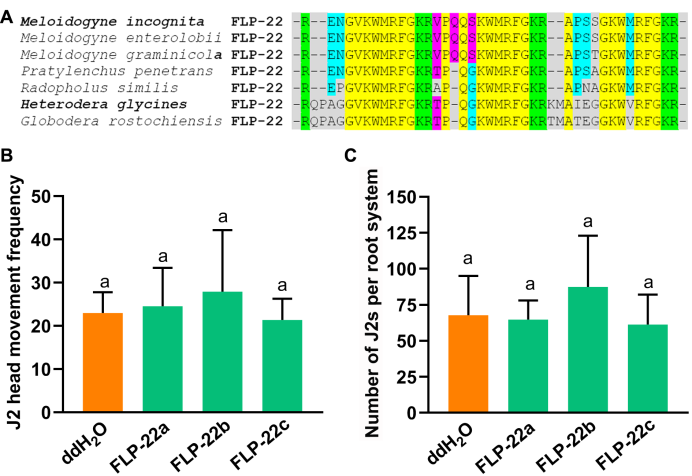


**Supplementary Figure 2. (A)** Multiple sequence alignments of partial FLP-22 peptides from plant-parasitic nematode species. Highly conserved di- and mono-basic cleavage sites residues are represented in green. Completely and partially conserved [amino acid residues](https://www.sciencedirect.com/topics/chemistry/amino-acid-residue) are highlighted in yellow and blue, respectively. (**B**) Effect of Hg-FLP-22 peptides on the frequency of J2s head movement of *Meloidogyne incognita*. Each value represents the mean head movement frequency ± SD from 60 individual worms. (**C)** Penetrated ability of Hg-FLP-22 peptides or ddH_2_O-treated nematodes in tomato roots. Each plant was inoculated with 200 J2s. The number of nematodes in tomato roots was counted at 24 h after inoculation (hai) through acid fuchsin staining. Data are the means ± SD from 8 plants, and are representative of three independent experiments. The same letters indicate no significant differences at *P* $˃$ 0.05 (Tukey’s HSD test).

## Supplementary Table1 List of primers used in this study.

| **Primer name** | **Primer sequence (5'→3')** | **Experimental purpose** | **Amplicon size (bp)** | **Annealing temperature (°C)** |
| --- | --- | --- | --- | --- |
| *Hg-flp-22* | F: AATGGTCGTCGTTCTGTCG  R: GTTTGCCTTGTGGGCGCTGC | Cloning of *Hg-flp-22* | 479 | 60 |
| *Hg-flp-22_qPCR* | F: GCTTCTCCGCCGACTTCA  R: GCCATTTTCCGCTTTCCGA | qRT-PCR | 220 | 60 |
| *Hg-GAPDH_ qPCR* | f: TCCAAGGCATAGAAAGACGACG  R: AACAAGTCATTGGACGGCATCA | qRT-PCR | 113 | 60 |
| *Hg-flp-22_ISH* | F: GCTTCTCCGCCGACTTCA  R: GATTGCCATTTTCCGCTTTC | RT-PCR for *In situ* hybridization | 224 | 60 |
| *Hg-flp-22_dsRNA* | **F: TAATACGACTCACTATAGGGAG**TTCTCCGCCGACTTCAC  **R: TAATACGACTCACTATAGGGAG**TCCCAAACCGTACCCAC | dsRNAs synthesis | 250 | 63 |
| *gfp_dsRNA* | **F:TAATACGACTCACTATAGGGAG**ATGGTGAGCAAGGGCGAG  **R:TAATACGACTCACTATAGGGAG**TTACTTGTACAGCTCGTCCATG | dsRNAs synthesis | 720 | 63 |

**Supplementary Table 2. Putative *H. glycine* *flp-22* gene and its homologues are conserved across the nematode phylum.** Interrogation of available nematode EST, genomic and transcriptomic datasets indicate at least 41 nematode species from clades 8, 9, 10 and 12 (Holterman et al., 2006) possess a homologue of the *Hg-flp-22* in phylum nematoda. Grey boxes indicate the presence of a *Hg-flp-22-*homologue, as identified via BLAST, in selected nematode species.

| **Clade** | **Nematode Species** | **Putative *Hg-flp-22* homologues** |
| --- | --- | --- |
| **8** | ***Ascaris suum*** |  |
|  | ***Brugia malayi*** |  |
|  | ***Brugia pahangi*** |  |
|  | ***Wuchereria bancrofti*** |  |
|  | ***Dirofilaris immitis*** |  |
|  | ***Onchocerca vovulus*** |  |
|  | ***Onchocerca ochengi*** |  |
|  | ***Loa loa*** |  |
|  | ***Litomosoides sigmodontis*** |  |
|  | ***Thelazia callipaeda*** |  |
|  | ***Dracunculus medinensis*** |  |
| **9** | ***Caenorhabditis elegans*** |  |
|  | ***Pristionchus pacificus*** |  |
|  | ***Pratylenchus penetrans*** |  |
|  | ***Caenorhabditis nigoni*** |  |
|  | ***Caenorhabditis briggsae*** |  |
|  | ***Caenorhabditis bovis*** |  |
|  | ***Caenorhabditis latens*** |  |
|  | ***Caenorhabditis japonica*** |  |
|  | ***Caenorhabditis brenneri*** |  |
|  | ***Caenorhabditis remanei*** |  |
|  | ***Necator americanus*** |  |
|  | ***Nippostrongylus brasilliensis*** |  |
|  | ***Ancylostoma canium*** |  |
|  | ***Ancylostoma ceylanicum*** |  |
|  | ***Ancylostoma duodenale*** |  |
|  | ***Anisakis simplex*** |  |
|  | ***Ostertagia circumcincta*** |  |
|  | ***Ostertagia ostertagi*** |  |
|  | ***Oesophagostomum dentatum*** |  |
|  | ***Oesophagostomum dentatum*** |  |
|  | ***Haemonchus contortus*** |  |
|  | ***Haemonchus placei*** |  |
|  | ***Teladorsagia circumcincta*** |  |
|  | ***Trichostrongylus colubriformis*** |  |
|  | ***Diploscapter pachys*** |  |
| **10** | ***Bursaphelenchus xylophilus*** |  |
|  | ***Parastrongyloides trichosuri*** |  |
|  | ***Strongyloides ratti*** |  |
|  | ***Strongyloides stercoralis*** |  |
|  | ***Steinernema carpocapsae*** |  |
| **12** | ***Angiostrongylus costaricensis*** |  |
|  | ***Meloidogyne hapla*** |  |
|  | ***Radopholus similis*** |  |
|  | ***Meloidogyne incognita*** |  |
|  | ***Meloidogyne graminicola*** |  |
|  | ***Meloidogyne enterolobii*** |  |
|  | ***Globodera pallida*** |  |
|  | ***Globodera rostochiensis*** |  |
|  | ***Deladenus siriddlcola*** |  |
|  | ***Dictyocaulus viviparus*** |  |
|  | ***Heligmosomoides polygyrus*** |  |
|  | ***Halicephalobus Panagrolaimidae*** |  |
|  | ***Toxocara canis*** |  |
